# Supplementary material for: Early Maladaptive Schemas and Schema Modes among People with Histories of Suicidality and the Possibility of a Universal Pattern: A Systematic Review
Source: Brain Sci. 2023 Aug 17;13(8):1216. doi: 10.3390/brainsci13081216 (PMC10452338; doi:10.3390/brainsci13081216)
Supplement: Supplementary file 1 [file brainsci-13-01216-s001.zip › Table S2. Research selection process.pdf]

Table S2: Research selection process.

ALL FOUND WITHIN DATABASES (DELETED REPETITIONS)

**PUBMED (19)**	
1.	Early maladaptive schemas and suicidal risk in inpatients with bipolar disorder.<br>Khosravani V, Mohammadzadeh A, Sharifi Bastan F, Amirinezhad A, Amini M.Psychiatry Res. 2019 Jan;271:351-359. doi: 10.1016/j.psychres.2018.11.067. Epub 2018 Dec 1.PMID: 30529318
2.	Early maladaptive schemas and suicidal risk in an Iranian sample of patients with obsessive-compulsive disorder.<br>Khosravani V, Sharifi Bastan F, Samimi Ardestani M, Jamaati Ardakani R.Psychiatry Res. 2017 Sep;255:441-448. doi: 10.1016/j.psychres.2017.06.080. Epub 2017 Jun 27.PMID: 28686949
3.	Association of parasuicidal behaviour to early maladaptive schemas and schema modes in patients with BPD: The Oulu BPD study.<br>Leppänen V, Vuorenmaa E, Lindeman S, Tuulari J, Hakko H.Personal Ment Health. 2016 Feb;10(1):58-71. doi: 10.1002/pmh.1304. Epub 2015 Sep 2.PMID: 26333517
4.	Early Maladaptive Schemas in Bipolar Disorder Patients With and Without Suicide Attempts.<br>Nilsson KK.J Nerv Ment Dis. 2016 Mar;204(3):236-9. doi: 10.1097/NMD.0000000000000451.PMID: 26919302
5.	The role of parental bonding and early maladaptive schemas in the risk of suicidal behavior repetition.<br>Dale R, Power K, Kane S, Stewart AM, Murray L.Arch Suicide Res. 2010;14(4):311-28. doi: 10.1080/13811118.2010.524066.PMID: 21082448
6.	Suicidal ideation and suicide attempts among university students in South Korea during the COVID-19 pandemic: the application of interpersonal-psychological theory and early maladaptive schema.<br>Ha J, Park D.PeerJ. 2022 Jul 27;10:e13830. doi: 10.7717/peerj.13830. eCollection 2022.PMID: 35915755
7.	Maladaptive schemas mediate poor parental attachment and suicidality in college students.<br>Langhinrichsen-Rohling J, Thompson K, Selwyn C, Finnegan H, Misra T.Death Stud. 2017 Jul;41(6):337-344. doi: 10.1080/07481187.2017.1280714. Epub 2017 Jan 12.PMID: 28080438
8.	Early Maladaptive Schemas in Eating Disordered Patients With or Without Non-Suicidal Self-Injury.<br>Pauwels E, Dierckx E, Schoevaerts K, Claes L.Eur Eat Disord Rev. 2016 Sep;24(5):399-405. doi: 10.1002/erv.2460. Epub 2016 Jun 28.PMID: 27349211

9.	Early maladaptive schemas associated with dimensional and categorical psychopathology in patients with borderline personality disorder.<br>Frías Á, Navarro S, Palma C, Farriols N, Aliaga F, Salvador A, Aluco E, Martínez B, Solves L.Clin Psychol Psychother. 2018 Jan;25(1):e30-e41. doi: 10.1002/cpp.2123. Epub 2017 Aug 17.PMID: 28833873
10.	The mediatory role of maladaptive schema modes between parental care and non-suicidal self-injury.<br>Saldias A, Power K, Gillanders DT, Campbell CW, Blake RA.Cogn Behav Ther. 2013;42(3):244-57. doi: 10.1080/16506073.2013.781671. Epub 2013 Jun 4.PMID: 23734870
11.	Early maladaptive schemas of emotional deprivation, social isolation, shame and abandonment are related to a history of suicide attempts among patients with major depressive disorders.<br>Ahmadpanah M, Astinsadaf S, Akhondi A, Haghighi M, Sadeghi Bahmani D, Nazaribadie M, Jahangard L, Holsboer-Trachsler E, Brand S.Compr Psychiatry. 2017 Aug;77:71-79. doi: 10.1016/j.comppsych.2017.05.008. Epub 2017 Jun 19.PMID: 28636896
12.	Borderline Personality Features in Students: the Predicting Role of Schema, Emotion Regulation, Dissociative Experience and Suicidal Ideation.<br>Sajadi SF, Arshadi N, Zargar Y, Mehrabizade Honarmand M, Hajjari Z.Int J High Risk Behav Addict. 2015 Jun 30;4(2):e20021. doi: 10.5812/ijhrba.20021v2. eCollection 2015 Jun.PMID: 26401490
13.	A new integrative model for the co-occurrence of non-suicidal self-injury behaviours and eating disorder symptoms.<br>Krug I, Arroyo MD, Giles S, Dang AB, Kiropoulos L, De Paoli T, Buck K, Treasure J, Fuller-Tyszkiewicz M.J Eat Disord. 2021 Nov 22;9(1):153. doi: 10.1186/s40337-021-00508-3.PMID: 34809723
14.	[Early maladaptive processes, depression and alexithymia in suicidal hospitalized adolescents].<br>Hirsch N, Hautekeete M, Kochman F.Encephale. 2001 Jan-Feb;27(1):61-70.PMID: 11294040
15.	Individual risk factors for physician boundary violations: the role of attachment style, childhood trauma and maladaptive beliefs.<br>MacDonald K, Sciolla AF, Folsom D, Bazzo D, Searles C, Moutier C, Thomas ML, Borton K, Norcross B.Gen Hosp Psychiatry. 2015 Jan-Feb;37(1):81-8. doi: 10.1016/j.genhosppsych.2014.09.001. Epub 2014 Sep 6.PMID: 25440724
16.	[Exploring Early Maladaptative Schema (EMS) in adults with bipolar disorder: A systematic review of the scientific literature].<br>Munuera C, Weil F, Minois I, Zanouy L, Gard S, Roux P, M'Bailara K.Encephale. 2020 Feb;46(1):65-77. doi: 10.1016/j.encep.2019.09.005. Epub 2019 Nov 22.PMID: 31767255
17.	[Effect of schema-focused therapy on depression, anxiety and maladaptive cognitive schemas in the elderly].<br>Kindynis S, Burlacu S, Louville P, Limosin F.Encephale. 2013 Dec;39(6):393-400. doi: 10.1016/j.encep.2013.04.002. Epub 2013 Jul 3.PMID: 23830011
18.	Scores on the suicide cognitions scale-revised (SCS-R) predict future suicide attempts among primary care patients denying suicide ideation and prior attempts.

	Bryan CJ, Thomsen CJ, Bryan AO, Baker JC, May AM, Allen MH.J Affect Disord. 2022 Sep 15;313:21-26. doi: 10.1016/j.jad.2022.06.070. Epub 2022 Jun 27.
19.	Core schemas and suicidality in a chronically traumatized population.<br>Dutra L, Callahan K, Forman E, Mendelsohn M, Herman J.J Nerv Ment Dis. 2008 Jan;196(1):71-4. doi: 10.1097/NMD.0b013e31815fa4c1.PMID: 18195645
**EBSCO (26)**	
20.	~~Early maladaptive schemas and suicidal risk in an Iranian sample of patients with obsessive-compulsive disorder.~~<br>~~Khosravani V; Sharifi Bastan F; Samimi Ardestani M; Jamaati Ardakani R, Psychiatry research [Psychiatry Res], ISSN: 1872-7123, 2017 Sep; Vol. 255, pp. 441-448; Publisher: Elsevier/North-Holland Biomedical Press; PMID: 28686949, Baza danych: MEDLINE~~
21.	Early maladaptive schemas are associated with increased suicidal risk among individuals with schizophrenia.<br>Azadi, Shahdokht; Khosravani, Vahid; Naragon-Gainey, Kristin; Bastan, Farangis Sharifi; Mohammadzadeh, Ali; Ghorbani, Fatemeh; International Journal of Cognitive Therapy, Vol 12(4), Dec, 2019 pp. 274-291. Publisher: Springer; [Journal Article], Baza danych: APA PsycInfo
22.	~~Early maladaptive schemas and suicidal risk in inpatients with bipolar disorder.~~<br>~~Khosravani V; Mohammadzadeh A; Sharifi Bastan F; Amirinezhad A; Amini M, Psychiatry research [Psychiatry Res], ISSN: 1872-7123, 2019 Jan; Vol. 271, pp. 351-359; Publisher: Elsevier/North-Holland Biomedical Press; PMID: 30529318, Baza danych: MEDLINE~~
23.	The role of unmet needs in self-harming behaviours.<br>Patterson, Allisha; Dissertation Abstracts International: Section B: The Sciences and Engineering, Vol 78(1-B)(E) Publisher: ProQuest Information & Learning; [Dissertation], Baza danych: APA PsycInfo
24.	~~Suicidal ideation and suicide attempts among university students in South Korea during the COVID-19 pandemic: the application of interpersonal-psychological theory and early maladaptive schema.~~<br>~~Ha J; Park D, PeerJ [PeerJ], ISSN: 2167-8359, 2022 Jul 27; Vol. 10, pp. e13830; Publisher: PeerJ Inc; PMID: 35915755, Baza danych: MEDLINE~~
25.	~~The role of parental bonding and early maladaptive schemas in the risk of suicidal behavior repetition.~~<br>~~Dale, Rosanna; Power, Kevin; Kane, Scott; Stewart, Alex Mitchell; Murray, Lindsey; Archives of Suicide Research, Vol 14(4), Oct, 2010 pp. 311-328. Publisher: Taylor & Francis; [Journal Article], Baza danych: APA PsycInfo~~
26.	~~Association of parasuicidal behaviour to early maladaptive schemas and schema modes in patients with BPD: The Oulu BPD study.~~<br>~~Leppänen, Virpi; Vuorenmaa, Elina; Lindeman, Sari; Tuulari, Jyrki; Hakko, Helinä; Personality and Mental Health, Vol 10(1), Feb, 2016 pp. 58-71. Publisher: John Wiley & Sons; [Journal Article], Baza danych: APA PsycInfo~~

27.	~~Early maladaptive schemas in bipolar disorder patients with and without suicide attempts.~~<br>~~Nilsson, Kristine Kahr; Journal of Nervous and Mental Disease, Vol 204(3), Mar, 2016 pp. 236-239. Publisher: Lippincott Williams & Wilkins; [Journal Article], Baza danych: APA PsycInfo~~
28.	Early maladaptive schemas and suicidal ideation in depressed patients.<br>Flink, N.; Lehto, S. M.; Koivumaa-Honkanen, H.; Viinamäki, H.; Ruusunen, A.; Valkonen-Korhonen, M.; Honkalampi, K.; The European Journal of Psychiatry, Vol 31(3), Jul-Sep, 2017 pp. 87-92. Publisher: Elsevier Science; [Journal Article], Baza danych: APA PsycInfo
29.	~~Early maladaptive schemas of emotional deprivation, social isolation, shame and abandonment are related to a history of suicide attempts among patients with major depressive disorders.~~<br>~~Ahmadpanah, Mohammad; Astinsadaf, Sommayyeh; Akhondi, Amineh; Haghighi, Mohammad; Sadeghi Bahmani, Dena; Nazaribadie, Marzieh; Jahangard, Leila; Holsboer-Trachsler, Edith; Brand, Serge; Comprehensive Psychiatry, Vol 77, Aug, 2017 pp. 71-79. Publisher: Elsevier Science; [Journal Article], Baza danych: APA PsycInfo~~
30.	~~Early Maladaptive Schemas in Eating Disordered Patients With or Without Non-Suicidal Self-Injury.~~<br>~~Pauwels E; Dierckx E; Schoevaerts K; Claes L, European eating disorders review : the journal of the Eating Disorders Association [Eur Eat Disord Rev], ISSN: 1099-0968, 2016 Sep; Vol. 24 (5), pp. 399-405; Publisher: Wiley; PMID: 27349211, Baza danych: MEDLINE~~
31.	A comparison of the early maladaptive schemas of suicidal and non-suicidal depressed patients with non-clinical sample.<br>Ahmadian, Masoomeh; Fata, Ladan; Asgharnezhad, Aliasghar; Malakooti, Kazem; Advances in Cognitive Science, Vol 10(4)[40], Win 2008 pp. 98. Publisher: Institute for Cognitive Science Studies; [Journal Article], Baza danych: APA PsycInfo
32.	Personality traits, early maladaptive schemas, and severity of nonsuicidal self-injury.<br>Arthurs, Sarah D.; Tan, Josephine C. H.; Psi Chi Journal of Psychological Research, Vol 22(3), Fal 2017 pp. 181-192. Publisher: Psi Chi National Office; [Journal Article], Baza danych: APA PsycInfo
33.	Erken dönem uyumsuz şemaların ergenlik dönemi depresif bozuklukla ilişkisi.<br>Relation between early maladaptive schemas of adolescents and depressive disorder. Gökçe, Sebla; Önal Sönmez, Arzu; Yusufoğlu, Canan; Yulaf, Yasemin; Adak, İbrahim; Anadolu Psikiyatri Dergisi, Vol 18(3), 2017 pp. 283-291. Publisher: Cumhuriyet University Faculty of Medicine; [Journal Article], Baza danych: APA PsycInfo
34.	~~Early maladaptive schemas associated with dimensional and categorical psychopathology in patients with borderline personality disorder.~~<br>~~Frías, Álvaro; Navarro, Sara; Palma, Carol; Farriols, Núria; Aliaga, Ferrán; Salvador, Ana; Aluco, Elena; Martínez, Bárbara; Solves, Laia; Clinical Psychology & Psychotherapy, Vol 25(1), Jan-Feb, 2018 pp. e30-e41. Publisher: John Wiley & Sons; [Journal Article], Baza danych: APA PsycInfo~~
35.	~~[Exploring Early Maladaptative Schema (EMS) in adults with bipolar disorder: A systematic review of the scientific literature].~~

	~~Munuera C; Weil F; Minois I; Zanouy L; Gard S; Roux P; M'Bailara K, L'Encephale [Encephale], ISSN: 0013-7006, 2020 Feb; Vol. 46 (1), pp. 65-77; Publisher: Masson; PMID: 31767255, Baza danych: MEDLINE~~
36.	~~Borderline Personality Features in Students: the Predicting Role of Schema, Emotion Regulation, Dissociative Experience and Suicidal Ideation.~~<br>~~Sajadi SF; Arshadi N; Zargar Y; Mehrabizade Honarmand M; Hajjari Z, International journal of high risk behaviors & addiction [Int J High Risk Behav Addict], ISSN: 2251-8711, 2015 Jun 30; Vol. 4 (2), pp. e20021; Publisher: Brieflands; PMID: 26401490, Baza danych: MEDLINE~~
37.	Influence du trouble bipolaire sur les facteurs cognitifs et affectifs: Étude dans une population de patients adultes libanais.<br>Influence of bipolar disorder on cognitive and emotional factors: Study in a population of Lebanese adult patients. Richa, Nathalie; Richa, Sami; Annales Médico-Psychologiques, Vol 171(5), Jun, 2013 pp. 295-299. Publisher: Elsevier Masson SAS; [Journal Article], Baza danych: APA PsycInfo
38.	Introduction to special issue: Cognition and emotion in borderline personality disorder.<br>Arntz, Arnoud; Journal of Behavior Therapy and Experimental Psychiatry, Vol 36(3), Sep, 2005 Special Issue: Cognition and Emotion in Borderline Personality Disorder. pp. 167-172. Publisher: Elsevier Science; [Editorial], Baza danych: APA PsycInfo
39.	~~Maladaptive schemas mediate poor parental attachment and suicidality in college students.~~<br>~~Langhinrichsen-Rohling J; Thompson K; Selwyn C; Finnegan H; Misra T, Death studies [Death Stud], ISSN: 1091-7683, 2017 Jul; Vol. 41 (6), pp. 337-344; Publisher: Taylor and Francis; PMID: 28080438, Baza danych: MEDLINE~~
40.	~~Core schemas and suicidality in a chronically traumatized population.~~<br>~~Dutra, Lissa; Callahan, Kelley; Forman, Evan; Mendelsohn, Michaela; Herman, Judith; Journal of Nervous and Mental Disease, Vol 196(1), Jan, 2008 pp. 71-74. Publisher: Lippincott Williams & Wilkins; [Journal Article], Baza danych: APA PsycInfo~~
41.	Suicidality. Newman, Cory F.; In: Psychological treatment of bipolar disorder. Johnson, Sheri L. (Ed); Leahy, Robert L. (Ed); Publisher: The Guilford Press; 2004, pp. 265-285. [Chapter], Baza danych: APA PsycInfo
42.	Symptômes reliés au diagnostic du trouble de personnalité limite à l'adolescence: Une recension systématique de la littérature.<br>Translated Title: Symptoms related to the diagnosis of borderline personality disorder in adolescence: A systematic review of the literature. Le Bœuf, Élodie; Sénéchal, Carole; Larivée, Serge; Annales Médico-Psychologiques, Vol 179(10), Dec, 2021 pp. 924-939. Publisher: Elsevier Masson SAS; [Journal Article], Baza danych: APA PsycInfo
43.	Socioenvironmental and cognitive risk and resources: Relations to mood and suicidality among inpatient adolescents.<br>Reinecke, Mark A.; DuBois, David L.; Journal of Cognitive Psychotherapy, Vol 15(3), Fal 2001 Special Issue: Developmental parameters for cognitive therapy with youth. pp. 195-222. Publisher: Springer Publishing; [Journal Article], Baza danych: APA PsycInfo
44.	~~Thérapie des schémas du sujet âgé : Impact sur la dépression, l'anxiété et les schémas cognitifs typiques.~~

	Translated Title: Effect of schema-focused therapy on depression, anxiety and maladaptive cognitive schemas in the elderly. Kindynis, S.; Burlacu, S.; Louville, P.; Limosin, F.; L'Encéphale: Revue de psychiatrie clinique biologique et thérapeutique, Vol 39(6), Dec, 2013 pp. 393-400. Publisher: Elsevier Masson SAS; [Journal Article], Baza danych: APA PsycInfo
45.	[Effect of schema-focused therapy on depression, anxiety and maladaptive cognitive schemas in the elderly].<br>Kindynis S; Burlacu S; Louville P; Limosin F, L'Encephale [Encephale], ISSN: 0013-7006, 2013 Dec; Vol. 39 (6), pp. 393-400; Publisher: Masson; PMID: 23830011, Baza danych: MEDLINE
**ScienceDirect (12)**	
46.	Early maladaptive schemas and suicidal risk in inpatients with bipolar disorder<br>Psychiatry Research1 December 2018, Vahid Khosravani, Ali Mohammadzadeh, Marziyeh Amini
47.	Early maladaptive schemas, suicidal ideation, and self-harm: A meta-analytic review<br>Journal of Affective Disorders Reports5 December 2020, Pamela Pilkington, Rita Younan, Amy Bishop
48.	Early maladaptive schemas and suicidal risk in an Iranian sample of patients with obsessive-compulsive disorder<br>Psychiatry ResearchSeptember 2017, Vahid Khosravani, Farangis Sharifi Bastan, Razieh Jamaati Ardakani
49.	Early maladaptive schemas and suicidal ideation in depressed patients<br>The European Journal of PsychiatryJuly–September 2017, N. Flink, S. M. Lehto, K. Honkalampi
50.	Emotion regulation as a mediator between early maladaptive schemas and non-suicidal self-injury in youth<br>Journal of Behavioral and Cognitive Therapy24 June 2022, Annemarie Nicol, Phillip S. Kavanagh, Anita S. Mak
51.	A Comparison of Relationship between Early Maladaptive Schemas with Depression Severity in Suicidal Group and Non-clinical Sample Procedia - Social and Behavioral Sciences9 July 2013, Farah darvishi, Mohamad ali rahmani, Morteza Rahbar
52.	L’exploration des Schémas Précoces Inadaptés (SPI) chez les personnes adultes atteintes de troubles bipolaires: une revue systématique de la littérature scientifique L'Encéphale22 November 2019, C. Munuera, F. Weil, K. M’Bailara
53.	Early maladaptive schemas of emotional deprivation, social isolation, shame and abandonment are related to a history of suicide attempts among patients with major depressive disorders Comprehensive Psychiatry August 2017, Mohammad Ahmadpanah, Sommayyeh Astinsadaf, Serge Brand
54.	Thérapie des schémas chez la personne âgée déprimée Journal de Thérapie Comportementale et CognitiveMarch 2013, Sophie Kindynis, Margaux René
55.	Symptômes reliés au diagnostic du trouble de personnalité limite à l’adolescence : une recension systématique de la littérature Annales Médico-psychologiques, revue psychiatrique22 September 2020, Élodie Le Bœuf, Carole Sénéchal, Serge Larivée

56.	~~Influence du trouble bipolaire sur les facteurs cognitifs et affectifs : étude dans une population de patients adultes libanais~~<br>~~Annales Médico-psychologiques, revue psychiatriqueJune 2013~~, ~~Nathalie Richa~~, ~~Sami Richa~~
57.	~~Thérapie des schémas du sujet âgé : impact sur la dépression, l'anxiété et les schémas cognitifs typiques L'EncéphaleDecember 2013~~; ~~S. Kindynis~~; ~~S. Burlacu~~; ~~F. Limosin~~
**Scopus (19)**	
58.	~~Suicidal ideation and suicide attempts among university students in South Korea during the COVID-19 pandemic: The application of interpersonal-psychological theory and early maladaptive schema~~<br>~~Ha, J., Park, D.~~
59.	Role of Early Maladaptive Schemas and Alexithymia in the Relationship Between Perceived Parenting Styles in Moroccan Psychoactive Substance Users<br>Karjouh, K., Azzaoui, F.-Z., Boulbaroud, S., Samlali, W.I., Ahami, A.
60.	A Novel Application of the Schema Therapy Mode Model for Social Anxiety Disorder: A Naturalistic Case Study<br>Penney, E.S., Norton, A.R.
61.	Search for a New Diagnostic Significance of the Young Questionnaire in Relation to Patients with Personality Disorders Showing a Tendency to Suicidal Behavior | Поиск новой диагностической значимости опросника Янга в отношении пациентов с расстройствами личности и склонностью к суицидальному поведению<br>Savenkova, V.I.
62.	The relationship between early maladaptive schemas and the functions of self-injurious behaviour in youth<br>Nicol, A., Mak, A.S., Murray, K., Kavanagh, P.S.
63.	~~Early maladaptive schemas, suicidal ideation, and self-harm: A meta-analytic review~~<br>~~Pilkington, P., Younan, R., Bishop, A.~~
64.	Healthy emotions, lower risk? The relationship between emotional states and violence risk among offenders with Cluster B personality disorders<br>Clercx, M., Keulen–de Vos, M.E., Beurskens, J.
65.	~~Exploring Early Maladaptative Schema (EMS) in adults with bipolar disorder: A systematic review of the scientific literature | L'exploration des Schémas Précoces Inadaptés (SPI) chez les personnes adultes atteintes de troubles bipolaires: une revue systématique de la littérature scientifique~~<br>~~Munuera, C., Weil, F., Minois, I., ...Roux, P., M'Bailara, K.~~
66.	~~Early Maladaptive Schemas Are Associated with Increased Suicidal Risk among Individuals with Schizophrenia~~<br>~~Azadi, S., Khosravani, V., Naragon-Gainey, K., ...Mohammadzadeh, A., Ghorbani, F.~~

67.	~~Early maladaptive schemas and suicidal risk in inpatients with bipolar disorder~~<br>~~Khosravani, V., Mohammadzadeh, A., Sharifi Bastan, F., Amirinezhad, A., Amini, M.~~
68.	~~Early maladaptive schemas associated with dimensional and categorical psychopathology in patients with borderline personality disorder~~<br>~~Frías, Á., Navarro, S., Palma, C., ...Martínez, B., Solves, L.~~
69.	~~Early maladaptive schemas and suicidal risk in an Iranian sample of patients with obsessive-compulsive disorder~~<br>~~Khosravani, V., Sharifi Bastan, F., Samimi Ardestani, M., Jamaati Ardakani, R.~~
70.	~~Early maladaptive schemas of emotional deprivation, social isolation, shame and abandonment are related to a history of suicide attempts among patients with major depressive disorders~~<br>~~Ahmadpanah, M., Astinsadaf, S., Akhondi, A., ...Holsboer-Trachsler, E., Brand, S.~~
71.	~~Early maladaptive schemas and suicidal ideation in depressed patients~~<br>~~Flink, N., Lehto, S.M., Koivumaa-Honkanen, H., ...Valkonen-Korhonen, M., Honkalampi, K.~~
72.	Early maladaptive schemas and level of depression in alcohol addicts<br>Jabłoński, M., Chodkiewicz, J.
73.	~~Association of parasuicidal behaviour to early maladaptive schemas and schema modes in patients with BPD: The Oulu BPD study~~<br>~~Leppänen, V., Vuorenmaa, E., Lindeman, S., Tuulari, J., Hakko, H.~~
74.	~~Early maladaptive schemas in bipolar disorder patients with and without suicide attempts~~<br>~~Nilsson, K.K.~~
75.	~~Borderline personality features in students: The predicting role of schema, emotion regulation, dissociative experience and suicidal ideation~~<br>~~Sajadi, S.F., Arshadi, N., Zargar, Y., Honarmand, M.M., Hajjari, Z.~~
76.	~~The role of parental bonding and early maladaptive schemas in the risk of suicidal behavior repetition~~<br>~~Dale, R., Power, K., Kane, S., Stewart, A.M., Murray, L.~~
**COCHRANE (3)**	
77.	Psychological interventions for antisocial personality disorder Simon Gibbon, Najat R Khalifa, Natalie H-Y Cheung, Birgit A Völlm, Lucy McCarthy
78.	Psychosocial interventions for conversion and dissociative disorders in adults Christina A Ganslev, Ole Jakob Storebø, Henriette E Callesen, Rachel Ruddy, Ulf Søgaard

79.	The effect of Acceptance and Commitment Therapy on experiential avoidance and emotion regulation in college students with interpersonal problems IRCT2015051922321N1https://trialsearch.who.int/Trial2.aspx?TrialID=IRCT2015051922321N1, 2016 | added to CENTRAL: 31 March 2019 | 2019 Issue 3

ANALYSIS BASED ON TITLES:

[1] regarding Non-Suicidal Self Injury

[2] not related to the topic

[3] regarding younger population

[4] foreign language

[5] systematic review, meta-analysis, book chapter

1.	Early maladaptive schemas and suicidal risk in inpatients with bipolar disorder.<br>Khosravani V, Mohammadzadeh A, Sharifi Bastan F, Amirinezhad A, Amini M.Psychiatry Res. 2019 Jan;271:351-359. doi: 10.1016/j.psychres.2018.11.067. Epub 2018 Dec 1.PMID: 30529318
2.	Early maladaptive schemas and suicidal risk in an Iranian sample of patients with obsessive-compulsive disorder.<br>Khosravani V, Sharifi Bastan F, Samimi Ardestani M, Jamaati Ardakani R.Psychiatry Res. 2017 Sep;255:441-448. doi: 10.1016/j.psychres.2017.06.080. Epub 2017 Jun 27.PMID: 28686949
3.	Association of parasuicidal behaviour to early maladaptive schemas and schema modes in patients with BPD: The Oulu BPD study.<br>Leppänen V, Vuorenmaa E, Lindeman S, Tuulari J, Hakko H.Personal Ment Health. 2016 Feb;10(1):58-71. doi: 10.1002/pmh.1304. Epub 2015 Sep 2.PMID: 26333517
4.	Early Maladaptive Schemas in Bipolar Disorder Patients With and Without Suicide Attempts.<br>Nilsson KK.J Nerv Ment Dis. 2016 Mar;204(3):236-9. doi: 10.1097/NMD.0000000000000451.PMID: 26919302
5.	The role of parental bonding and early maladaptive schemas in the risk of suicidal behavior repetition.<br>Dale R, Power K, Kane S, Stewart AM, Murray L.Arch Suicide Res. 2010;14(4):311-28. doi: 10.1080/13811118.2010.524066.PMID: 21082448
6.	Suicidal ideation and suicide attempts among university students in South Korea during the COVID-19 pandemic: the application of interpersonal-psychological theory and early maladaptive schema.<br>Ha J, Park D.PeerJ. 2022 Jul 27;10:e13830. doi: 10.7717/peerj.13830. eCollection 2022.PMID: 35915755
7.	Maladaptive schemas mediate poor parental attachment and suicidality in college students.<br>Langhinrichsen-Rohling J, Thompson K, Selwyn C, Finnegan H, Misra T.Death Stud. 2017 Jul;41(6):337-344. doi: 10.1080/07481187.2017.1280714. Epub 2017 Jan 12.PMID: 28080438

8.[1]	Early Maladaptive Schemas in Eating Disordered Patients With or Without Non-Suicidal Self-Injury.<br>Pauwels E, Dierckx E, Schoevaerts K, Claes L.Eur Eat Disord Rev. 2016 Sep;24(5):399-405. doi: 10.1002/erv.2460. Epub 2016 Jun 28.PMID: 27349211
9.	Early maladaptive schemas associated with dimensional and categorical psychopathology in patients with borderline personality disorder.<br>Frías Á, Navarro S, Palma C, Farriols N, Aliaga F, Salvador A, Aluco E, Martínez B, Solves L.Clin Psychol Psychother. 2018 Jan;25(1):e30-e41. doi: 10.1002/cpp.2123. Epub 2017 Aug 17.PMID: 28833873
10.[1,2]	The mediatory role of maladaptive schema modes between parental care and non-suicidal self-injury.<br>Saldias A, Power K, Gillanders DT, Campbell CW, Blake RA.Cogn Behav Ther. 2013;42(3):244-57. doi: 10.1080/16506073.2013.781671. Epub 2013 Jun 4.PMID: 23734870
11.	Early maladaptive schemas of emotional deprivation, social isolation, shame and abandonment are related to a history of suicide attempts among patients with major depressive disorders.<br>Ahmadpanah M, Astinsadaf S, Akhondi A, Haghighi M, Sadeghi Bahmani D, Nazaribadie M, Jahangard L, Holsboer-Trachsler E, Brand S.Compr Psychiatry. 2017 Aug;77:71-79. doi: 10.1016/j.comppsych.2017.05.008. Epub 2017 Jun 19.PMID: 28636896
12.	Borderline Personality Features in Students: the Predicting Role of Schema, Emotion Regulation, Dissociative Experience and Suicidal Ideation.<br>Sajadi SF, Arshadi N, Zargar Y, Mehrabizade Honarmand M, Hajjari Z.Int J High Risk Behav Addict. 2015 Jun 30;4(2):e20021. doi: 10.5812/ijhrba.20021v2. eCollection 2015 Jun.PMID: 26401490
13.[1,2]	A new integrative model for the co-occurrence of non-suicidal self-injury behaviours and eating disorder symptoms.<br>Krug I, Arroyo MD, Giles S, Dang AB, Kiropoulos L, De Paoli T, Buck K, Treasure J, Fuller-Tyszkiewicz M.J Eat Disord. 2021 Nov 22;9(1):153. doi: 10.1186/s40337-021-00508-3.PMID: 34809723
14.[3,4]	[Early maladaptive processes, depression and alexithymia in suicidal hospitalized adolescents].<br>Hirsch N, Hautekeete M, Kochman F.Encephale. 2001 Jan-Feb;27(1):61-70.PMID: 11294040
15.[2]	Individual risk factors for physician boundary violations: the role of attachment style, childhood trauma and maladaptive beliefs.<br>MacDonald K, Sciolla AF, Folsom D, Bazzo D, Searles C, Moutier C, Thomas ML, Borton K, Norcross B.Gen Hosp Psychiatry. 2015 Jan-Feb;37(1):81-8. doi: 10.1016/j.genhosppsych.2014.09.001. Epub 2014 Sep 6.PMID: 25440724
16.[5]	[Exploring Early Maladaptative Schema (EMS) in adults with bipolar disorder: A systematic review of the scientific literature].<br>Munuera C, Weil F, Minois I, Zanouy L, Gard S, Roux P, M'Bailara K.Encephale. 2020 Feb;46(1):65-77. doi: 10.1016/j.encep.2019.09.005. Epub 2019 Nov 22.PMID: 31767255
17.[2]	[Effect of schema-focused therapy on depression, anxiety and maladaptive cognitive schemas in the elderly].

	~~Kindynis S, Burlacu S, Louville P, Limosin F.Encephale. 2013 Dec;39(6):393-400. doi: 10.1016/j.encep.2013.04.002. Epub 2013 Jul 3.PMID: 23830011~~
18.[2]	~~Scores on the suicide cognitions scale-revised (SCS-R) predict future suicide attempts among primary care patients denying suicide ideation and prior attempts.~~<br>~~Bryan CJ, Thomsen CJ, Bryan AO, Baker JC, May AM, Allen MH.J Affect Disord. 2022 Sep 15;313:21-26. doi: 10.1016/j.jad.2022.06.070. Epub 2022 Jun 27.~~
19.	Core schemas and suicidality in a chronically traumatized population.<br>Dutra L, Callahan K, Forman E, Mendelsohn M, Herman J.J Nerv Ment Dis. 2008 Jan;196(1):71-4. doi: 10.1097/NMD.0b013e31815fa4c1.PMID: 18195645
20.	**Early maladaptive schemas** are associated with increased **suicidal risk** among individuals with schizophrenia.<br>Azadi, Shahdokht; Khosravani, Vahid; Naragon-Gainey, Kristin; Bastan, Farangis Sharifi; Mohammadzadeh, Ali; Ghorbani, Fatemeh; International Journal of Cognitive Therapy, Vol 12(4), Dec, 2019 pp. 274-291. Publisher: Springer; [Journal Article], Baza danych: APA PsycInfo
21.	The role of unmet needs in self-harming behaviours.<br>Patterson, Allisha; Dissertation Abstracts International: Section B: The Sciences and Engineering, Vol 78(1-B)(E) Publisher: ProQuest Information & Learning; [Dissertation], Baza danych: APA PsycInfo
22.	**Early maladaptive schemas** and **suicidal ideation** in depressed patients.<br>Flink, N.; Lehto, S. M.; Koivumaa-Honkanen, H.; Viinamäki, H.; Ruusunen, A.; Valkonen-Korhonen, M.; Honkalampi, K.; The European Journal of Psychiatry, Vol 31(3), Jul-Sep, 2017 pp. 87-92. Publisher: Elsevier Science; [Journal Article], Baza danych: APA PsycInfo
~~23.~~[4]	~~A comparison of the **early maladaptive schemas** of **suicidal** and non-**suicidal** depressed patients with non-clinical sample.~~<br>~~Ahmadian, Masoomeh; Fata, Ladan; Asgharnezhad, Aliasghar; Malakooti, Kazem; Advances in Cognitive Science, Vol 10(4)[40], Win 2008 pp. 98. Publisher: Institute for Cognitive Science Studies; [Journal Article], Baza danych: APA PsycInfo~~
24.	A Comparison of Relationship between Early Maladaptive Schemas with Depression Severity in Suicidal Group and Non-clinical Sample Procedia - Social and Behavioral Sciences9 July 2013, Farah darvishi, Mohamad ali rahmani, Morteza Rahbar
25.[1,2]	~~Personality traits, **early maladaptive schemas**, and severity of nonsuicidal self-injury.~~<br>~~Arthurs, Sarah D.; Tan, Josephine C. H.; Psi Chi Journal of Psychological Research, Vol 22(3), Fal 2017 pp. 181-192. Publisher: Psi Chi National Office; [Journal Article], Baza danych: APA PsycInfo~~
26.[3,4]	~~Erken dönem uyumsuz şemaların ergenlik dönemi depresif bozuklukla ilişkisi.~~<br>~~Relation between **early maladaptive schemas** of adolescents and depressive disorder. Gökçe, Sebla; Önal Sönmez, Arzu; Yusufoğlu, Canan; Yulaf, Yasemin; Adak, İbrahim; Anadolu Psikiyatri Dergisi, Vol 18(3), 2017 pp. 283-291. Publisher: Cumhuriyet University Faculty of Medicine; [Journal Article], Baza danych: APA PsycInfo~~

~~27.[2]~~	~~Influence du trouble bipolaire sur les facteurs cognitifs et affectifs: Étude dans une population de patients adultes libanais. Influence of bipolar disorder on cognitive and emotional factors: Study in a population of Lebanese adult patients. ki, Nathalie; Richa, Sami; Annales Médico-Psychologiques, Vol 171(5), Jun, 2013 pp. 295-299. Publisher: Elsevier Masson SAS; [Journal Article], Baza danych: APA PsycInfo~~
28.[2]	Introduction to special issue: Cognition and emotion in borderline personality disorder.<br>Arntz, Arnoud; Journal of Behavior Therapy and Experimental Psychiatry, Vol 36(3), Sep, 2005 Special Issue: Cognition and Emotion in Borderline Personality Disorder. pp. 167-172. Publisher: Elsevier Science; [Editorial], Baza danych: APA PsycInfo
~~29.[5]~~	~~**Suicidality**. Newman, Cory F.; In: Psychological treatment of bipolar disorder. Johnson, Sheri L. (Ed); Leahy, Robert L. (Ed); Publisher: The Guilford Press; 2004, pp. 265-285. [Chapter], Baza danych: APA PsycInfo~~
30.[3]	~~Symptômes reliés au diagnostic du trouble de personnalité limite à l'adolescence: Une recension systématique de la littérature. Translated Title: Symptoms related to the diagnosis of borderline personality disorder in adolescence: A systematic review of the literature. Le Bœuf, Élodie; Sénéchal, Carole; Larivée, Serge; Annales Médico-Psychologiques, Vol 179(10), Dec, 2021 pp. 924-939. Publisher: Elsevier Masson SAS; [Journal Article], Baza danych: APA PsycInfo~~
31.[3]	~~Socioenvironmental and cognitive **risk** and resources: Relations to mood and **suicidality** among inpatient adolescents. Reinecke, Mark A.; DuBois, David L.; Journal of Cognitive Psychotherapy, Vol 15(3), Fal 2001 Special Issue: Developmental parameters for cognitive therapy with youth. pp. 195-222. Publisher: Springer Publishing; [Journal Article], Baza danych: APA PsycInfo~~
~~32.[5]~~	~~Early maladaptive schemas, suicidal ideation, and self-harm: A meta-analytic review~~<br>~~Journal of Affective Disorders Reports5 December 2020~~, ~~Pamela Pilkington~~, ~~Rita Younan, Amy Bishop~~
33.[1,3]	~~Emotion regulation as a mediator between early maladaptive schemas and non-suicidal self-injury in youth~~<br>~~Journal of Behavioral and Cognitive Therapy24 June 2022~~, ~~Annemarie Nicol~~, ~~Phillip S. Kavanagh~~, ~~Anita S. Makcom~~
34.[2]	~~Thérapie des schémas chez la personne âgée déprimée~~<br>~~Journal de Thérapie Comportementale et CognitiveMarch 2013~~, ~~Sophie Kindynis~~, ~~Margaux René~~
35.[2]	~~*Role of Early Maladaptive Schemas and Alexithymia in the Relationship Between Perceived Parenting Styles in Moroccan Psychoactive Substance Users*~~<br>~~Karjouh, K., Azzaoui, F.-Z., Boulbaroud, S., Samlali, W.I., Ahami, A.~~
36.[2]	~~*A Novel Application of the Schema Therapy Mode Model for Social Anxiety Disorder: A Naturalistic Case Study*~~<br>~~Penney, E.S., Norton, A.R.~~

~~37.~~[4]	~~*Search for a New Diagnostic Significance of the Young Questionnaire in Relation to Patients with Personality Disorders Showing a Tendency to Suicidal Behavior | Поиск новой диагностической значимости опросника Янга в отношении пациентов с расстройствами личности и склонностью к суицидальному поведению*~~<br>~~*Savenkova, V.I.*~~
38.[1,3]	~~*The relationship between early maladaptive schemas and the functions of self-injurious behaviour in youth*~~<br>~~*Nicol, A., Mak, A.S., Murray, K., Kavanagh, P.S.*~~
39.[2]	~~*Healthy emotions, lower risk? The relationship between emotional states and violence risk among offenders with Cluster B personality disorders Clercx, M., Keulen-de Vos, M.E., Beurskens, J.*~~
40.[2]	~~Psychological interventions for antisocial personality disorder Simon Gibbon, Najat R Khalifa, Natalie H-Y Cheung, Birgit A Völlm, Lucy McCarthy~~
41.[2]	~~Psychosocial interventions for conversion and dissociative disorders in adults Christina A Ganslev, Ole Jakob Storebø, Henriette E Callesen, Rachel Ruddy, Ulf Søgaard~~
42.[2]	~~The effect of Acceptance and Commitment Therapy on experiential avoidance and emotion regulation in college students with interpersonal problems IRCT2015051922321N1 https://trialsearch.who.int/Trial2.aspx?TrialID=IRCT2015051922321N1, 2016 | added to CENTRAL: 31 March 2019 | 2019 Issue 3~~

ANALYSIS BASED ON ABSTRACTS:

[1] regarding younger population

[2] no Early Maladaptive Schemas or Schema Modes measures

1.	Early maladaptive schemas and suicidal risk in inpatients with bipolar disorder.<br>Khosravani V, Mohammadzadeh A, Sharifi Bastan F, Amirinezhad A, Amini M.Psychiatry Res. 2019 Jan;271:351-359. doi: 10.1016/j.psychres.2018.11.067. Epub 2018 Dec 1.PMID: 30529318<br><br>The present study aimed to assess the associations of early maladaptive schemas (EMSs) and clinical factors (hypomanic/manic and depressive symptoms) with suicidal risk (current suicidal ideation and lifetime suicide attempts) in inpatients with bipolar disorder (BD). One hundred inpatients with BD completed the Young Schema Questionnaire-Short Form (YSQ-SF), the Bipolar Depression Rating Scale (BDRS), the Young Mania<br>Rating Scale (YMRS), and the Beck Scale for Suicide Ideation (BSSI). 59% of patients had lifetime suicide attempts and 59% showed high suicidal risk (BSSI ≥ 6). BD patients with lifetime suicide attempts had higher scores on the entitlement and social isolation schemas, depression, and hypomanic/manic symptoms than those without such attempts. Patients with high suicidal risk had higher levels of depressive and hypomanic/manic symptoms as well as some EMSs than those without high suicidal risk. Logistic regression analyses revealed that hypomanic/manic symptoms as well as the entitlement and defectiveness schemas were significantly associated with current suicidal ideation. Also, the entitlement and social isolation schemas were associated with lifetime suicide attempts. These results suggest that the entitlement, social isolation, and defectiveness schemas may relate to suicidal risk in patients with BD.
2.	Early maladaptive schemas and suicidal risk in an Iranian sample of patients with obsessive-compulsive disorder.<br>Khosravani V, Sharifi Bastan F, Samimi Ardestani M, Jamaati Ardakani R.Psychiatry Res. 2017 Sep;255:441-448. doi: 10.1016/j.psychres.2017.06.080. Epub 2017 Jun 27.PMID: 28686949<br><br>There are few studies on suicidal risk and its related factors in patients diagnosed with obsessive-compulsive disorder (OCD). This study investigated the associations of early maladaptive schemas, OC symptom dimensions, OCD severity, depression and anxiety with suicidality (i.e., suicidal ideation and suicide attempts) in OCD patients. Sixty OCD outpatients completed the Scale for Suicide Ideation (SSI), the Young Schema QuestionnaireShort Form (YSQ-SF), the Yale-Brown Obsessive Compulsive Scale (Y-BOCS), the Dimensional ObsessiveCompulsive Scale (DOCS) and the Depression Anxiety Stress Scales (DASS-21). 51.7% of patients had lifetime suicide attempts and 75% had suicidal ideation. OCD patients with lifetime suicide attempts exhibited significantly higher scores on

	early maladaptive schemas than those without such attempts. Logistic regression analysis revealed that the mistrust/abuse schema and the OC symptom dimension of unacceptable thoughts explained lifetime suicide attempts. The mistrust/abuse schema, unacceptable thoughts and depression significantly predicted suicidal ideation. These findings indicated that the mistrust/abuse schema may contribute to high suicidality in OCD patients. Also, patients suffering from unacceptable thoughts need to be assessed more carefully for warning signs of suicide.
3.	Association of parasuicidal behaviour to early maladaptive schemas and schema modes in patients with BPD: The Oulu BPD study. Leppänen V, Vuorenmaa E, Lindeman S, Tuulari J, Hakko H.Personal Ment Health. 2016 Feb;10(1):58-71. doi: 10.1002/pmh.1304. Epub 2015 Sep 2.PMID: 26333517<br><br>The present study aimed to examine which early maladaptive schemas (EMSs) and schema modes emerged in parasuicidal and non-parasuicidal patients with BPD participating in the Oulu BPD study. The patients' EMSs were assessed using the Young Schema Questionnaire, and schema modes using the Young Atkinson Mode Inventory. Sixty patients with BPD responded to both the schema and schema mode questionnaires; of these, 46 (76.7%) fulfilled the criteria for parasuicidality. In BPD patients with parasuicidality, the EMSs of emotional deprivation, abandonment/instability, mistrust/abuse and social isolation were the most prevalent, and the schema modes of vulnerable child, angry child, detached protector and compliant surrender were prominent. In patients without parasuicidality, the schema modes of healthy adult and happy child were the most prevalent. Significant correlations were observed between the schema modes of detached protector, vulnerable child, punitive parent and angry child and almost every EMS in BPD patients with parasuicidality. Our preliminary findings suggest that associations between certain EMSs and schema modes to parasuicidality in BPD patients may provide valuable information when planning and implementing their treatment.
4.	Early Maladaptive Schemas in Bipolar Disorder Patients With and Without Suicide Attempts.<br>Nilsson KK.J Nerv Ment Dis. 2016 Mar;204(3):236-9. doi: 10.1097/NMD.0000000000000451.PMID: 26919302<br><br>Patients with bipolar disorder (BD) are at an increased risk of attempted and completed suicide. To elucidate the beliefs and assumptions associated with suicidality in BD, the present study compared BD patients with and without a history of suicide attempt in terms of early maladaptive schemas (EMSs). The sample consisted of 49 remitted BD patients who completed the Young Schema Questionnaire-Short Version. Information on suicide attempts was obtained through interviews combined with medical records. Compared with BD patients without suicide attempts, the BD patients with suicide attempts scored significantly higher on 3 EMSs: social isolation, practical incompetence, and entitlement. The findings suggest that specific EMSs may be implicated in suicidal behaviors in BD. These results have implications for the assessment and treatment of suicidality in BD.

5.	The role of parental bonding and early maladaptive schemas in the risk of suicidal behavior repetition.<br>Dale R, Power K, Kane S, Stewart AM, Murray L.Arch Suicide Res. 2010;14(4):311-28. doi: 10.1080/13811118.2010.524066.PMID: 21082448<br><br>The current study examined the role of perceived parental bonding and early maladaptive schemas in suicidal behavior. Participants completed measures of perceived parental bonding; schemas; risk of repeating suicidal behavior; anxiety; and depression following their presentation at Accident and Emergency with suicidal behavior. A suicidal behavior group (n = 60) differed from a comparison clinical (n = 46) and non-clinical (n = 48) group on measures of early maladaptive schemas, anxiety, and depression. No significant difference was noted between the suicidal behavior group and the comparison clinical group on a measure of parental bonding. Within the suicidal behavior group, significant associations were indicated between perceived parental bonding and risk of repetition of suicidal behavior; and early maladaptive schemas and risk of repetition of suicidal behavior. Early maladaptive schemas were found to mediate the relationship between perceived parental bonding and risk of repetition of suicidal behavior, with schemas of Social Alienation and Defectiveness/Shame offering mediator roles. The findings of the current study emphasize the complexities of suicidal behavior and factors that are associated with suicidal behavior. Although causality cannot be assumed, the findings highlight the importance and inter-relationships of not only perceived early experiences, but of underlying schemas in relation to suicidal behavior.
6.	Suicidal ideation and suicide attempts among university students in South Korea during the COVID-19 pandemic: the application of interpersonal-psychological theory and early maladaptive schema.<br>Ha J, Park D.PeerJ. 2022 Jul 27;10:e13830. doi: 10.7717/peerj.13830. eCollection 2022.PMID: 35915755<br><br>Background This study examined the application of interpersonal-psychological theory and early maladaptive schema of suicidal ideation and suicide attempts in South Korean university students. Methods In this cross-sectional study, data from 367 university students were surveyed using the Interpersonal Needs Questionnaire, Early Maladaptive Schema, Suicide Ideation Scale, and the Acquired Capability for Suicide Scale. Data were collected between June 21 and July 21, 2021. Results University students' interpersonal needs and early maladaptive schema were significantly associated with suicidal ideation, and influencing suicide attempts. The acquired capability for suicide moderated the relationship between suicidal ideation and attempts. Conclusions In suicide prevention programs for university students, it is critical to consider their interpersonal needs and early maladaptive schema, and the acquired capability for suicide, to prevent suicidal ideation and attempts among them.
7.	Maladaptive schemas mediate poor parental attachment and suicidality in college students.<br>Langhinrichsen-Rohling J, Thompson K, Selwyn C, Finnegan H, Misra T.Death Stud. 2017 Jul;41(6):337-344. doi: 10.1080/07481187.2017.1280714. Epub 2017 Jan 12.PMID: 28080438

	In college-aged adults (n = 766), the transition to adulthood may aggravate risk factors for suicidal behavior such as poor parental attachment and maladaptive self-schemas. Because poor parental attachment may facilitate developing maladaptive self-related schemas, this study was designed to determine whether specific maladaptive schemas mediate the relation between poor parental attachment and college students' suicide proneness and ideation. Findings supported this hypothesis. Defectiveness and emotional deprivation schemas, which are consistent with "perceived burdensomeness" and "thwarted belonging," may be important intervention targets for suicide prevention programs. The ongoing role of parental attachment during early adult development is highlighted.
8.	Early maladaptive schemas associated with dimensional and categorical psychopathology in patients with borderline personality disorder.<br>Frías Á, Navarro S, Palma C, Farriols N, Aliaga F, Salvador A, Aluco E, Martínez B, Solves L.Clin Psychol Psychother. 2018 Jan;25(1):e30-e41. doi: 10.1002/cpp.2123. Epub 2017 Aug 17.PMID: 28833873<br><br>**Objective**<br><br>To ascertain the foundations for a schema-focused therapy model for the treatment of borderline personality disorder.<br><br>**Methods**<br><br>The sample consisted of 102 borderline personality disorder outpatients. Dimensional psychopathology was assessed using the Scale for Suicidal Ideation, the Aggression Questionnaire, and the Symptom CheckList Revised. Categorical psychopathology was measured using the structured clinical interview for Diagnostic and Statistical Manual of Mental Disorders, Fourth Edition Axis I Disorders, Patient Edition. Stepwise linear/logistic multiple regression analyses were used to determine the predictive role of the schema domains tested by the Young Schema Questionnaire on both types of psychopathology. Receiver operating characteristic curves were calculated for those binary outcomes.<br><br>**Results**<br><br>Regarding dimensional psychopathology, disconnection/rejection predicted greater suicidal ideation (β = .39, p = .002), physical/overt aggressiveness (β = .27, p = .05), and psychotic-like symptoms, such as paranoid ideation (β = .35, p = .003). Other-directedness predicted greater anger/inner aggressiveness (β = .22, p = .05) and internalizing symptoms, such as phobic anxiety (β = .39, p = .001).

	Regarding categorical psychopathology, disconnection/rejection significantly predicted the presence of lifetime comorbidities with eating disorders (adjusted odds ratio [AOR] = 1.12, 95% CI = 0.99–1.24) and posttraumatic stress disorder (AOR = 1.2, 95% CI = 1.04–1.3), resulting in a good balance of sensitivity/specificity, respectively (.97/.96 and .88/.89). Other-directedness significantly predicted the absence of lifetime comorbidity with substance-use disorders (AOR = .74, 95% CI = 0.57–0.95). These relationships remained significant after controlling for confounders (e.g., comorbidity with other personality disorders, clinical global severity).<br><br>**Conclusions**<br><br>Two schema domains, disconnection/rejection and other-directedness, were directly associated with dimensional and categorical psychopathology among borderline personality disorder patients. These findings provide further information about the foundations and target interventions when implementing schema-focused therapy on this population.
9.	Early maladaptive schemas of emotional deprivation, social isolation, shame and abandonment are related to a history of suicide attempts among patients with major depressive disorders.<br>Ahmadpanah M, Astinsadaf S, Akhondi A, Haghighi M, Sadeghi Bahmani D, Nazaribadie M, Jahangard L, Holsboer-Trachsler E, Brand S.Compr Psychiatry. 2017 Aug;77:71-79. doi: 10.1016/j.comppsych.2017.05.008. Epub 2017 Jun 19.PMID: 28636896<br><br>**Background**<br><br>Patients with psychiatric disorders have an exceptionally high risk of completed or attempted suicide. This holds particularly true for patients with major depressive disorders. The aim of the present study was to explore whether patients with major depressive disorders (MDD) and a history of suicide attempts differed in their early maladaptive schemas from patients with MDD but without such a history or from healthy controls.<br><br>**Method**<br><br>Ninety participants took part in the study. Of these, 30 were patients with MDD who had made a recent suicide attempt; 30 were patients with MDD but no suicide attempts, and 30 were gender- and age-matched healthy controls. Participants completed questionnaires covering socio-demographic characteristics and the Young Schema Questionnaire (YSQ- RE2R) to assess early maladaptive schemas. Experts rated patients' MDD with the Montgomery–Asberg Depression Rating Scale.

	**Results**<br><br>Patients did not differ in experts' ratings of symptoms of depression. Compared to healthy controls, patients with MDD recorded higher scores on maladaptive schemas such as recognition seeking, negativity/pessimism, and insufficient self-control. Compared to patients without suicide attempts and healthy controls, those who had made a suicide attempt had higher scores on dimensions such as failure, mistrust, emotional inhibition, social isolation, and abandonment/instability.<br><br>**Conclusion**<br><br>Compared to healthy controls, patients with MDD had more pronounced maladaptive schemas, but this was more marked in patients with a history of suicide attempts. The results suggest that suicide attempts and poorer psychological functioning are related.
10.[1]	~~Borderline Personality Features in Students: the Predicting Role of Schema, Emotion Regulation, Dissociative Experience and Suicidal Ideation.~~<br>~~Sajadi SF, Arshadi N, Zargar Y, Mehrabizade Honarmand M, Hajjari Z.Int J High Risk Behav Addict. 2015 Jun 30;4(2):e20021. doi: 10.5812/ijhrba.20021v2. eCollection 2015 Jun.PMID: 26401490~~<br><br>~~**Background:** Numerous studies have demonstrated that early maladaptive schemas, emotional dysregulation are supposed to be the defining core of borderline personality disorder. Many studies have also found a strong association between the diagnosis of borderline personality and the occurrence of suicide ideation and dissociative symptoms.~~<br><br>~~**Objectives:** The present study was designed to investigate the relationship between borderline personality features and schema, emotion regulation, dissociative experiences and suicidal ideation among high school students in Shiraz City, Iran.~~<br><br>~~**Patients and methods:** In this descriptive correlational study, 300 students (150 boys and 150 girls) were selected from the high schools in Shiraz, Iran, using the multi-stage random sampling. Data were collected using some instruments including borderline personality feature scale for children, young schema questionnaire-short form, difficulties in emotion-regulation scale (DERS), dissociative experience scale and beck suicide ideation scale. Data were analyzed using the Pearson correlation coefficient and multivariate regression analysis.~~

	~~**Results:** The results showed a significant positive correlation between schema, emotion regulation, dissociative experiences and suicide ideation with borderline personality features. Moreover, the results of multivariate regression analysis suggested that among the studied variables, schema was the most effective predicting variable of borderline features (P < 0.001).~~<br><br>~~**Conclusions:** The findings of this study are in accordance with findings from previous studies, and generally show a meaningful association between schema, emotion regulation, dissociative experiences, and suicide ideation with borderline personality features.~~
11.	Core schemas and suicidality in a chronically traumatized population.<br>Dutra L, Callahan K, Forman E, Mendelsohn M, Herman J.J Nerv Ment Dis. 2008 Jan;196(1):71-4. doi: 10.1097/NMD.0b013e31815fa4c1.PMID: 18195645<br><br>The Young Schema Questionnaire (YSQ) has been demonstrated to tap into core beliefs, or maladaptive schemas, of clinical populations. This study used the YSQ to investigate maladaptive schemas of 137 chronically traumatized patients seeking outpatient psychiatric treatment and to assess whether specific schemas might be associated with suicide risk in this population. Participants completed a modified version of the YSQ-S (short form), post-traumatic diagnostic scale, dissociative experiences scale and self-harm and risk behaviors questionnaire-revised at treatment intake. Significant correlations were found between most YSQ scales and the post-traumatic diagnostic scale, and between all YSQ scales and the dissociative experiences scale. Suicide risk variables were most highly correlated with the social isolation/alienation, defectiveness/shame and failure YSQ scales, suggesting that these schemas may mark individuals at particularly high risk for suicidal ideation and suicide attempts. These results offer important implications for the assessment and treatment of high-risk traumatized patients.
12.	**Early maladaptive schemas** are associated with increased **suicidal risk** among individuals with schizophrenia.<br>Azadi, Shahdokht; Khosravani, Vahid; Naragon-Gainey, Kristin; Bastan, Farangis Sharifi; Mohammadzadeh, Ali; Ghorbani, Fatemeh; International Journal of Cognitive Therapy, Vol 12(4), Dec, 2019 pp. 274-291. Publisher: Springer; [Journal Article], Baza danych: APA PsycInfo<br><br>Early maladaptive schemas (EMSs) are a dysfunctional cognitive pattern that can result from maladaptive functioning during childhood. EMSs are broad patterns of memories, emotions, cognitions, and bodily sensations related to the self and others. The aims of this study were to evaluate EMSs among individuals with schizophrenia and to evaluate the relations of EMSs and clinical factors (e.g., depression, positive, and negative symptoms of psychosis) to suicidal risk (e.g., current suicidal ideation, lifetime suicide attempts). Eighty-two inpatients with schizophrenia completed the Young Schema Questionnaire-Short Form (YSQ-SF), the Beck Depression Inventory-II

	(BDI-II), the Beck Scale for Suicide Ideation (BSSI), and the Positive and Negative Syndrome Scale (PANSS). Individuals with schizophrenia who had attempted suicide (relative to those who had not attempted suicide) had significantly higher EMSs, current suicidal ideation, and a family history of suicide attempts. Logistic regression analysis revealed that the emotional deprivation schema, positive symptoms, and depression were significantly associated with current suicidal ideation. In addition, emotional deprivation was significantly associated with lifetime suicide attempts. These findings suggest that the emotional deprivation schema, positive symptoms, and depression may be related to suicide in individuals with schizophrenia.
13.[2]	~~The role of unmet needs in self-harming behaviours.~~<br>~~Patterson, Allisha; Dissertation Abstracts International: Section B: The Sciences and Engineering, Vol 78(1-B)(E) Publisher: ProQuest Information & Learning; [Dissertation], Baza danych: APA PsycInfo~~<br><br>~~Suicide and self-mutilation are widespread societal issues, making them an important area of research. The interpersonal theory of suicide (ITS) and Shneidman's theory of psychache have each garnered substantial support in suicide research; however, to date, there have been no studies that combine the theories in hopes of creating a better model of suicide. There has also been very limited research that has applied these theories to self-mutilation, a behaviour highly correlated with suicide. It is expected that these two theories are compatible given that they both focus on unmet needs which create an undesirable state, and they both suggest that an additional factor is necessary for unmet needs to create suicidality. The current research also contributes theoretically to the literature by investigating previously unexplored possible antecedents to suicide and self-mutilation, namely adverse childhood experiences and early maladaptive schemas (unmet needs from childhood that influence an individual's interpretation of the world). Data were collected from two distinct populations: 428 university undergraduates and a community sample of 533 individuals. Participants completed questionnaires assessing the criterion measures of self-mutilation and suicidality and the psychological predictors of psychache, thwarted belongingness, perceived burdensomeness, and acquired capability for suicide. Analyses revealed that, for both the university and community samples, the ITS model (interaction of thwarted belonging, perceived burdensomeness and acquired capability) and psychache each added unique variance to the prediction of suicide ideation, motivation, preparation, and non-suicidal self-injury. A combined model, however, was not better able to predict a past suicide attempt. Further, contrary to expectation, the ITS-self-harm relationship was not mediated by psychache. Explanations for these findings are provided. Overall, these results provide important theoretical and practical contributions to the literature suggesting the utility of a new, blended model of suicide. Limitations and future directions are discussed.~~
14.	**Early maladaptive schemas** and **suicidal ideation** in depressed patients.<br>Flink, N.; Lehto, S. M.; Koivumaa-Honkanen, H.; Viinamäki, H.; Ruusunen, A.; Valkonen-Korhonen, M.; Honkalampi, K.; The European Journal of Psychiatry, Vol 31(3), Jul-Sep, 2017 pp. 87-92. Publisher: Elsevier Science; [Journal Article], Baza danych: APA PsycInfo

	Background and objectives<br>Suicidal ideation is a key risk factor for suicidal behaviour among depressed individuals. To explore underlying cognitive patterns associated with suicidal ideation, the present study compared early maladaptive schemas (EMSs) among psychiatric outpatients in treatment for major depressive disorder with and without current suicidal ideation.<br>Methods<br>The sample consisted of 79 depressed patients who responded to the background questionnaire and completed the Young Schema Questionnaire short form-extended, 21-item Beck Depression Inventory and Beck Hopelessness Scale.<br>Results<br>Patients with suicidal ideation were more maladaptive in respect to the majority of EMSs compared to those without. After controlling for the concurrent depressive symptom severity and hopelessness ‘Vulnerability to Harm or Illness’ EMS, which concerns catastrophising beliefs, remained a predictor for suicidal ideation.
~~15.[1]~~	~~A Comparison of Relationship between Early Maladaptive Schemas with Depression Severity in Suicidal Group and Non-clinical Sample Procedia - Social and Behavioral Sciences9 July 2013, Farah darvishi. Mohamad ali Rahmani, Morteza Rahbar~~<br><br>~~The aim of this study was to compare the relationship between between the content of early maladaptive schema and depression severity in suicidal patients and non-clinical. Sampling method was available of including 101 participants of suicidal patient and108 non-clinical participants who have been selected with random sampling method The young schema questioner short form version (75 items) and The Beck Depression Inventory-II were used. Significant relationship was found between, early maladaptive schemas with depression severity in both groups. Based on hierarchical regression analyses defectiveness/shame, failure, entitlement/grandiosity in suicidal group predicted depression severity and predictor schemas in non-clinical group were defectiveness/shame, entitlement/grandiosity, vulnerability to harm, and unrelenting standard. Analysis on z fishers didn’t indicate any significant difference for comparison relationships between of two groups.~~
